# Replication Publication

**DOI:** 10.1371/journal.pbio.0030327

**Published:** 2005-09-13

**Authors:** Mark Patterson, Lon Cardon

In December 2003, *PLoS Biology* published a research article reporting evidence of an association between a gene called *GAD2* and susceptibility to obesity (DOI: 10.1371/journal.pbio.0000068). Although the genetic data were suggestive rather than conclusive, the editors and reviewers supported publication because unambiguous evidence in association studies is notoriously difficult to obtain and obesity is such a major burden on public health. Enthusiasm was heightened because *GAD2* lies in a region of human Chromosome 10 that is thought to contain a gene influencing obesity, and *GAD2* itself is involved in the synthesis of a neurotransmitter implicated in the regulation of food intake. Publication would allow others to test just how important the connection between *GAD2* and obesity might be—and the enticing prospect was that insight into the role of *GAD2* in this disease might lead ultimately to new therapeutic approaches.

In this issue of *PLoS Biology*, we publish a follow-up to the original study, from a separate team of researchers based at the University of California at San Francisco (DOI: 10.1371/journal.pbio.0030315). Swarbrick et al. looked at different and much larger patient populations and analyzed variation within *GAD2* more comprehensively, but disappointingly, the new study was not able to replicate the initial finding.

Despite the negative conclusions, however, the reviewers were no less enthusiastic about the publication of this paper than the first. One of the reasons for this is related to a problem that has beset the field of complex disease genetics for several years—a tendency towards the publication of studies that show an association.

Diseases are termed complex when their etiology is influenced by many factors, both genetic and environmental. These diseases include obesity, diabetes, mental illness, and many more common ailments. But finding the genes that are involved with these diseases is an extremely difficult problem: individual genes have only a limited effect, which means that large patient populations need to be studied; specific genes might have effects only in certain ethnic groups; and statistical artifacts can be hard to eliminate. These and other problems mean that success stories are few and far between, whereas false leads have been plentiful. High-profile journals, in particular, have therefore tended to publish the positive results, whereas negative data often end up in specialist literature or, much worse, don't get published at all.

Because the identification of a complex disease gene is so difficult, the first positive report is rarely definitive—replication studies that use independent populations with sample sizes large enough to detect the expected effects are necessary to bolster (or undermine) the case. A bias towards publishing positive results might therefore perpetuate the false impression that a gene is indeed associated with a disease. In a field this complex, such bias is entirely unhelpful.

Replication studies are therefore vital to the field of human genetics, and at *PLoS Biology*, we've taken the view that well-designed, and high-powered replication studies can be just as worthy of publication in the journal as the initial finding of a genetic association—and PLoS's community journal *PLoS Genetics* takes a similar view. The new paper on *GAD2* demonstrates this point—the study was judged by the reviewers to be sufficiently large and well conducted to provide a robust test of the hypothesis that variation in *GAD2* is involved—above a certain minimum effect—in susceptibility to obesity. The negative conclusion suggests that researchers now need to consider possible reasons for the variability in results such as sample or phenotype differences, or else examine the possibility that other variants in the Chromosome 10 region might be involved in obesity. It might ultimately be that the initial finding simply does not hold in larger samples. That replication papers are important for the field is supported by the publication of another large study in type 2 diabetes (DOI: 10.1371/journal.pbio.0000020), which included replication data, and has already been cited 28 times.

Would editorial opinion of the paper by Swarbrick et al. have been any different if the result had been positive? We think not, because the first study left sufficient room for doubt, and a positive result could have been just as significant. In general, then, we will judge a replication paper on its merits, and consider whether the submitted work provides a major advance on the previous genetic findings.

But *PLoS Biology* and *PLoS Genetics* are both highly selective journals. By themselves they cannot solve the problem, and will only publish the most significant association studies—whether the results are positive or negative. Furthermore, the accumulation of genetic association data is likely only to accelerate in the coming years, thanks to major collaborative efforts such as the SNP Consortium (http://snp.cshl.org) and the International HapMap Project (http://www.hapmap.org). Public availability of these rich resources will stimulate genome-wide efforts to map the genes underlying any number of complex disorders. Data will abound, and they need to be published and also made publicly available. Most of the individual studies will not provide definitive evidence of associations, but as the data build, meta-analyses will become more and more informative.

PLoS is currently exploring the possibility of providing further publication opportunities to extend the venues that are available for association studies. As with all PLoS articles, the data and the associated papers would be freely available to read and reanalyze. With the introduction of more such open-access publications and with support from the community, it would be possible to eliminate bias in the publication of association studies for good.

